# Antineutrophilic cytoplasmic antibody-associated vasculitis with hypocomplementemia has a higher incidence of serious organ damage and a poor prognosis

**DOI:** 10.1097/MD.0000000000004871

**Published:** 2016-09-16

**Authors:** Shoichi Fukui, Naoki Iwamoto, Masataka Umeda, Ayako Nishino, Yoshikazu Nakashima, Tomohiro Koga, Shin-ya Kawashiri, Kunihiro Ichinose, Yasuko Hirai, Mami Tamai, Hideki Nakamura, Tomoki Origuchi, Shuntaro Sato, Atsushi Kawakami

**Affiliations:** a Department of Immunology and Rheumatology; b Department of Public Health; c Department of Rehabilitation Sciences, Unit of Translational Medicine, Nagasaki University Graduate School of Biomedical Sciences; d Nagasaki University Hospital Clinical Research Center, Nagasaki, Japan..

**Keywords:** complement, diffuse alveolar hemorrhage, hypocomplementemia, prognosis, relapse, thrombotic microangiopathy

## Abstract

A relationship between antineutrophilic cytoplasmic antibody (ANCA)-associated vasculitis (AAV) and complement has been shown, and complement has an important role in the pathogenesis of AAV. The clinical characteristics of AAV with hypocomplementemia still remain unclear.

We conducted an observational study of 81 patients with AAV (median onset age 71 years; 58% female). Using medical records, we analyzed the patients’ baseline variables, laboratory data, clinical symptoms, and therapeutic outcomes after treatments including episodes of relapses, initiation of dialysis, and death. We defined hypocomplementemia as the state in which at least one of the following was lower than the lower limit of the normal range: complement 3 (C3), complement 4 (C4), and total complement activity (CH50).

Sixteen patients (20%) had hypocomplementemia at their diagnosis of AAV. Compared to the AAV patients without hypocomplementemia (n = 65), those with hypocomplementemia had significantly higher rates of the occurrence of skin lesions (8 [50%] vs. 8 [12%], *P* = 0.002), diffuse alveolar hemorrhage (DAH) (6 [38%] vs. 5 [8%], *P* = 0.006), and thrombotic microangiopathy (TMA) (3 [19%] vs. 0 [0%], *P* = 0.007). The AAV patients with hypocomplementemia had significantly lower platelet levels (16.5 × 10^4^ vs. 24.9 × 10^4^ cells/μL, *P* = 0.023) compared to those without hypocomplementemia. More positive immune complex deposits in renal biopsy specimens were seen in the AAV patients with hypocomplementemia than in those without hypocomplementemia (4 [80%] vs. 2 [18%], *P* = 0.036). Assessed by a log-rank test, hypocomplementemia at disease onset was significantly associated with death (*P* = 0.033).

Hypocomplementemia in AAV at the disease onset was a risk factor for the serious organ damage, and a life prognostic factor. It is thus very important to pay attention to the levels of complement at the diagnosis of AAV.

## Abstract 2

Supplemental Digital Content is available in the text

## 1 Introduction

Antineutrophilic cytoplasmic antibody (ANCA)-associated vasculitis (AAV) is known as a systemic vasculitis with unknown etiology. AAV is classified as pauci-immune small vessel vasculitis,^[1]^ but mild glomerular tuft staining for immunoglobulins and/or complement is sometimes found by immunofluorescence in the glomerulonephritis region.^[2]^

A relationship between AAV and complement was recently demonstrated; complement has an important role in the pathogenesis of AAV.^[3]^ For example, in vitro, an alternative complement pathway was activated by ANCA-activated neutrophils,^[4]^ and in animal models, murine anti-myeloperoxidase (MPO) IgG-induced disease was prevented by the blockade of alternative complement pathway activation.^[5]^ Little information about the clinical features of AAV with hypocomplementemia in humans has been available. A clinical study of a small number of patients revealed that hypocomplementemia was associated with the patient prognosis and with a poor renal prognosis.^[6]^

Considering the important role of complement in AAV as revealed by experimental research, we suspected that hypocomplementemia might be related to clinical characteristics of AAV such as organ involvement, drug response, and prognosis. In this study, we attempted to clarify the clinical characteristics of AAV patients with hypocomplementemia.

## 2 Patients and methods

### 2.1 Patients

We retrospectively reviewed the cases of all patients who were newly diagnosed with AAV between April 2000 and June 2015 at Nagasaki University Hospital, Nagasaki, Japan. All patients were diagnosed as having AAV based on the Chapel Hill Consensus Conference criteria^[1]^ and the European Medicines Agency algorithm.^[7]^ The patients’ diagnoses included all types of AAV including eosinophilic granulomatous polyangiitis (EGPA), granulomatous polyangiitis (GPA), microscopic polyangiitis (MPA), and renal limited vasculitis (RLV).

**Figure 1:**
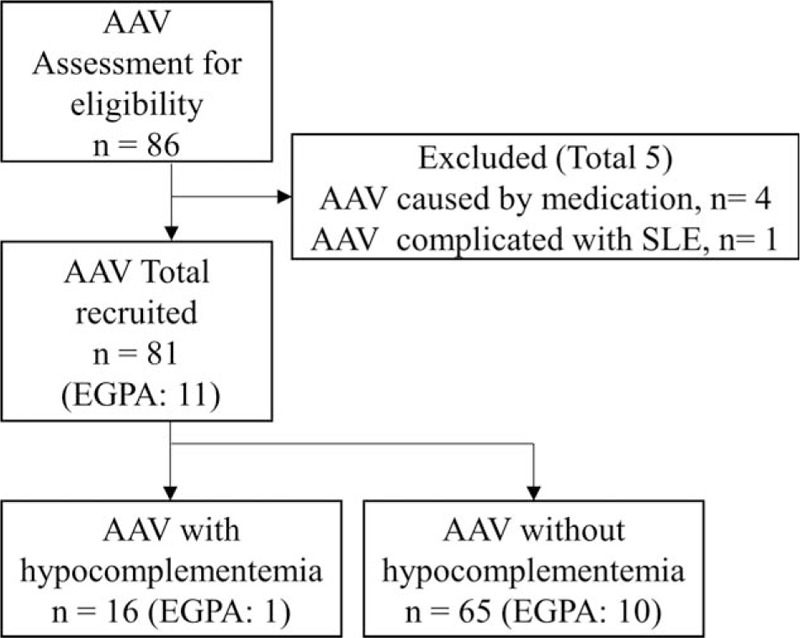
Flow diagram (81 patients with AAV were included in the study). AAV = antineutrophilic cytoplasmic antibody-associated vasculitis, EGPA = eosinophilic granulomatous polyangiitis.

Four AAV patients whose AAV was suspected to have been caused by medication were excluded. One patient who fulfilled the 1997 American College of Rheumatology classification criteria^[8]^ and the 2012 Systemic Lupus International Collaborating Clinics classification criteria^[9]^ for systemic lupus erythematosus (SLE) was excluded. A final total of 81 patients with AAV were included in the study (Fig. 1). This study was approved by the institutional review board (Institutional Review Board of Nagasaki University Hospital [NU15072753]). Informed consent was obtained from all patients.

### 2.2 Data collection

Using the patients’ medical records, we collected the demographic and clinical characteristics including symptoms and laboratory data at diagnosis, plus the treatments for AAV and therapeutic outcomes including relapse episodes, the initiation of dialysis, and death. We defined fever as persistent body temperature >38°C. Body weight loss was defined as a reduction in the patient's body weight by >5 kg during the first 6 months before the diagnosis of AAV. We defined skin disease as erythema or purpura. Lesions of the ear, nose, or throat associated with AAV were diagnosed by board-certified otolaryngologists of The Oto-Rhino-Laryngological Society of Japan.

Diffuse alveolar hemorrhage (DAH) was diagnosed based on minor or major hemoptysis and/or respiratory insufficiency together with at least 1 positive result on one of the following compatible complementary diagnostic tests: x-ray, computed tomography scan, and bronchoalveolar lavage.^[10]^ We diagnosed thrombotic microangiopathy (TMA) based on guidelines for the diagnosis and management of thrombotic thrombocytopenic purpura and other thrombotic microangiopathies.^[11]^ Rapidly progressive glomerulonephritis (RPGN) was diagnosed based on the following criteria: hematuria, proteinuria, or urine casts; estimated glomerular filtration rate <60 mL/min/1.73m^2^; and elevated C-reactive protein or erythrocyte sedimentation rate, for suspicion of RPGN, and the worsening of renal function over a period of several weeks and hematuria, proteinuria, or urine casts suggesting glomerulonephritis.^[12]^

We defined hypocomplementemia as the state in which at least one of the following was lower than the lower limit of the normal range: complement 3 (C3) (normal range 86–160 mg/dL), complement 4 (C4) (normal range 17–45 mg/dL), or total complement activity (CH50) (normal range 25–48 CH50/mL). C3 and C4 were determined by immunonephelometry and CH50 was measured according to Mayer method. The group of patients with hypocomplementemia was defined as the patients who had hypocomplementemia at disease onset. The group of patients without hypocomplementemia was defined as the patients who did not have hypocomplementemia at disease onset. Hematuria was defined as 2+ or more by urine analysis using a testing strip. Positive immune complex deposits in renal biopsy specimens were defined as 1+ or more of the glomerular immunofluorescence findings for at least of one of following: IgG, IgA, IgM, C3, C4, and C1q.

Pathological findings were assessed by board-certified pathologists of The Japanese Society of Pathology. Remission was defined as a Birmingham Vasculitis Activity Score, version 3 (BVAS), of 0 (scores range from 0 to 63, with higher scores indicating more active disease).^[13]^ We defined relapse as the reappearance or worsening of disease with a BVAS >0 and the involvement of at least one major organ, a life-threatening manifestation, or both.^[14]^

### 2.3 Statistical analysis

Baseline characteristics including demographics, hematologic data, serum markers, and pathological findings were compared between the patients with hypocomplementemia and those without hypocomplementemia. Variables were described with the use of frequencies for categorical variables and with the median and interquartile range (IQR) for quantitative variables. The association between variables was assessed using Fisher exact test for categorical variables and Wilcoxon rank sum test for quantitative variables.

The nonrelapse survival rate after remission and the probability of survival were estimated by the Kaplan–Meier method. The associations between the nonrelapse survival rate after remission, the probability of survival, and hypocomplementemia were evaluated by the log-rank test. All tests were 2-sided, and *P* values <0.05 were considered significant. All statistical analyses were performed using JMP Statistical Software, ver. 11 (SAS Institute, Cary, NC).

## 3 Results

### 3.1 Patient characteristics

Eighty-one patients with AAV (11 EGPA, 14 GPA, 53 MPA, and 3 renal-limited vasculitis) were included in this study. Table 1 shows the demographic and clinical characteristics of the total patient series and the patients with (n = 16) and without (n = 65) hypocomplementemia. All of the patients were Japanese. The median onset age was 71 years. Forty-seven patients (58%) were female.

**Table 1:**
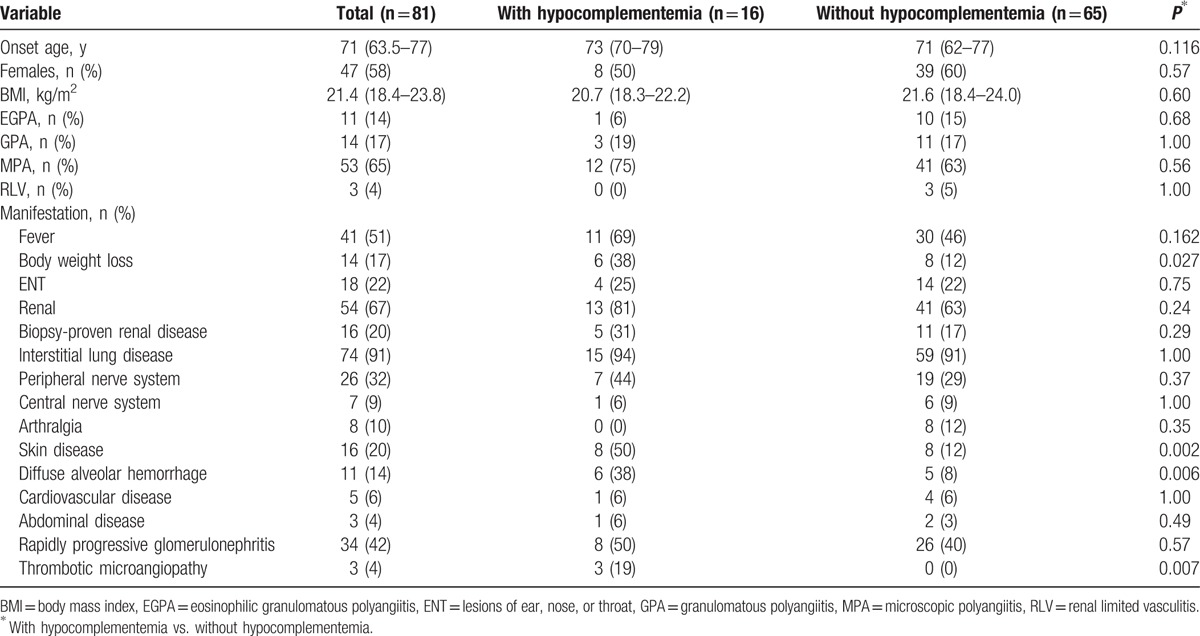
Demographic and clinical characteristics of total patients and patients with and without hypocomplementemia.

Sixteen patients (20%) had hypocomplementemia at the diagnosis of AAV. Compared to the AAV patients without hypocomplementemia, those with hypocomplementemia had significantly higher rates of the occurrence of skin lesions (8 [50%] vs. 8 [12%], *P* = 0.002), DAH (6 [38%] vs. 5 [8%], *P* = 0.006), and TMA (3 [19%] vs. 0 [0%], *P* = 0.007), respectively.

### 3.2 Laboratory data

Table 2 shows the laboratory data of the total patient series and the data of the patients with and without hypocomplementemia. The detailed level of each complement is illustrated in the Supplemental Figure. Compared to the AAV patients without hypocomplementemia, those with hypocomplementemia had significantly lower platelet levels (16.5 × 10^4^ vs. 24.9 × 10^4^ cells/μL, *P* = 0.023). The numbers of positive MPO-ANCA patients with AAV and that of positive PR3-ANCA patients with AAV were 68 (84%) and 8 (10%), respectively. The titers of MPO-ANCA and PR3-ANCA were not significantly different between the patients with and without hypocomplementemia (Suppl. Fig). Significantly more positive immune complex deposits in renal biopsy specimens were seen in the AAV patients with hypocomplementemia than in those without hypocomplementemia (4 [80%] vs. 2 [18%], *P* = 0.036).

**Table 2:**
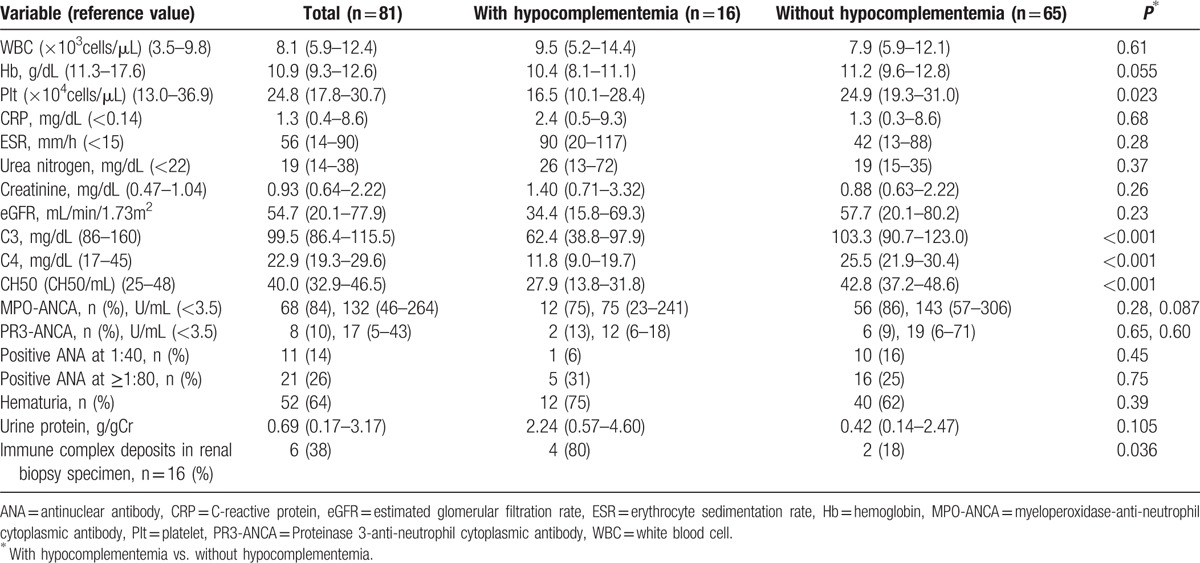
Laboratory data of total patients and the patients with and without hypocomplementemia.

### 3.3 Treatments

The median initial dose of prednisolone was 40 mg/day. There was no significant difference in the initial dose of prednisolone between the patients with and without hypocomplementemia. As shown in Table 3, other than prednisolone, there were no significant differences regarding treatments.

**Table 3:**
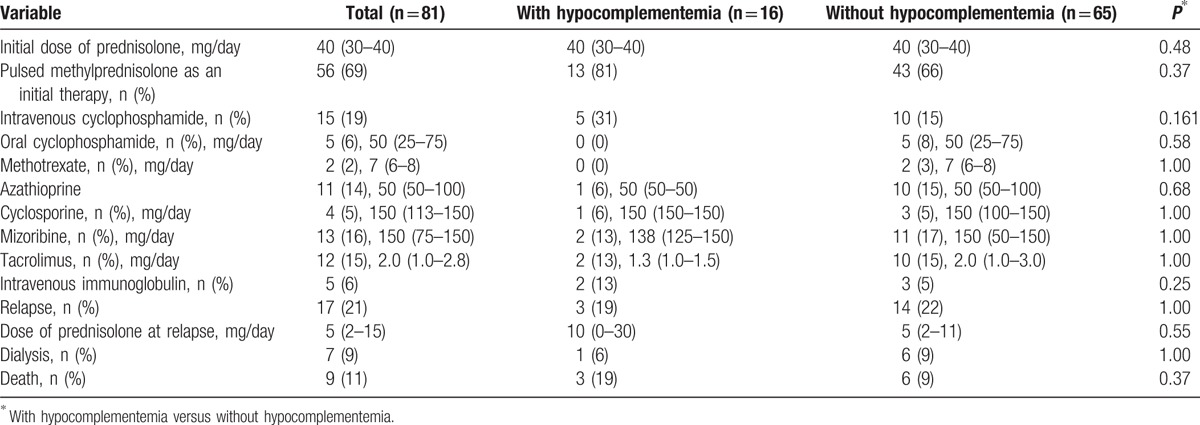
Treatments and outcomes of total patients and patients with and without hypocomplementemia.

### 3.4 Outcomes

**Figure 2:**
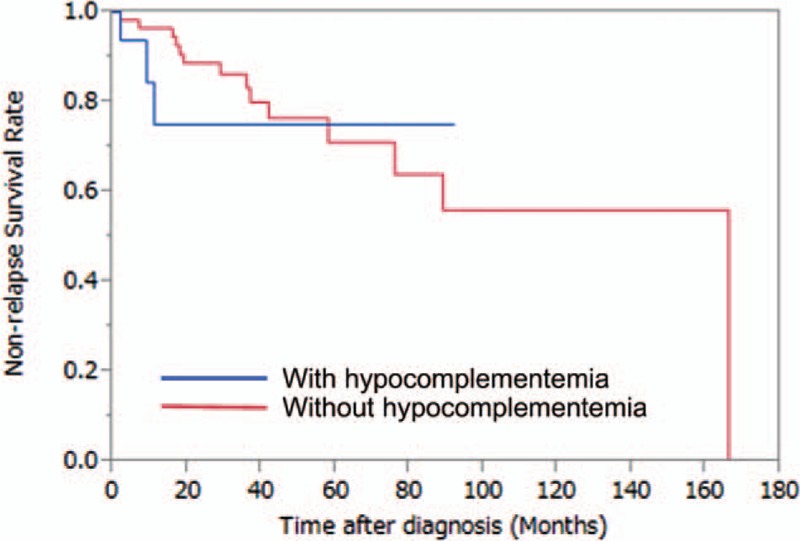
Hypocomplementemia at disease onset was not associated with subsequent relapses after remission (*P* = 0.27).

**Figure 3:**
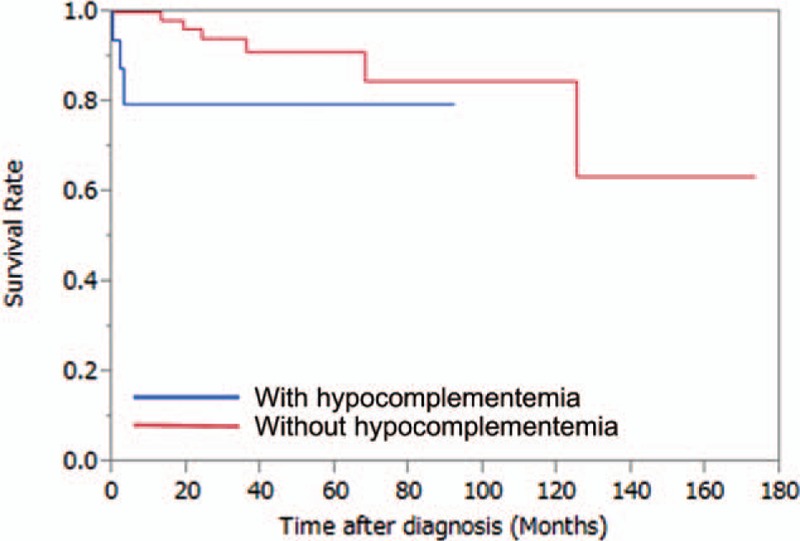
Hypocomplementemia at disease onset was significantly associated with death (*P* = 0.033).

Table 3 also summarizes the patient outcomes. Of the total group of 81 patients, 17 patients (21%) had relapses during follow-up. Hypocomplementemia at disease onset was not associated with subsequent relapses after remission as assessed by a log-rank test using the Kaplan–Meier method (*P* = 0.27) (Fig. 2). In contrast, hypocomplementemia at disease onset was significantly associated with death (*P* = 0.033) as assessed by a log-rank test using the Kaplan–Meier method (Fig. 3).

For all 7 patients needing dialysis, the initiation of dialysis was performed within 1 month after the diagnosis of AAV.

### 3.5 Results excluding the EGPA patients

**Figure 4:**
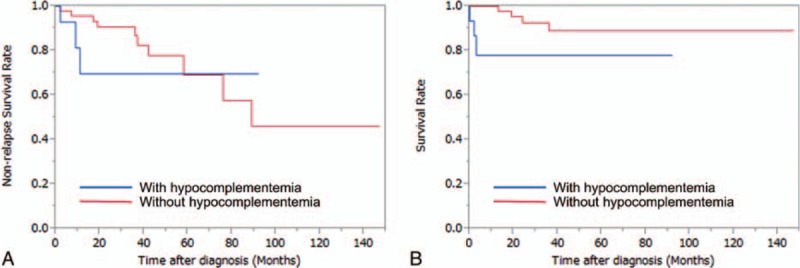
Analysis of data excluding eosinophilic granulomatous polyangiitis. (A) Hypocomplementemia at disease onset was not associated with subsequent relapses after remission (*P* = 0.31). (B) Hypocomplementemia at disease onset was significantly associated with death (*P* = 0.046).

We show results excluding those of the 11 EGPA patients (Table 4, Fig. 4) because EGPA is different from other types of AAV in its clinical manifestations and may have other pathogeneses even in the presence of ANCA. One patient with EGPA had hypocomplementemia at disease onset. The data excluding that of the EGPA patients also showed more skin lesions, DAH, TMA, skin lesions, and poor prognosis in the AAV patients with hypocomplementemia.

**Table 4:**
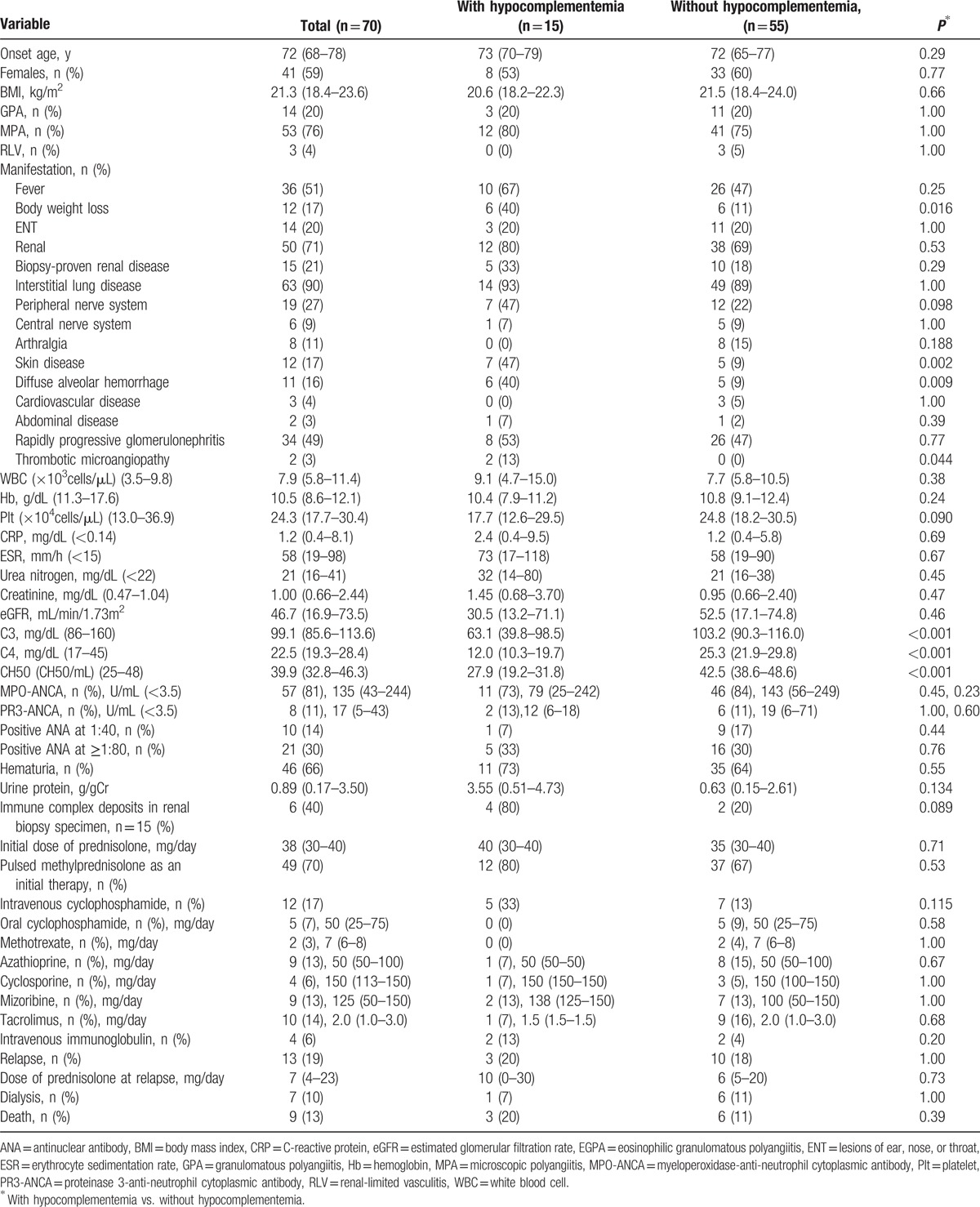
Demographic and clinical characteristics, laboratory data, treatments, and outcomes of total patients and patients with and without hypocomplementemia excluding EGPA.

### 3.6 Change in the levels of complement by treatment

We were able to collect the data of serial measurements of complements at both disease onset and remission from 11 of the 16 patients with hypocomplementemia from medical records, but only the complement levels at disease onset were available in the remaining 5 patients. Seven of the 11 patients for whom the data of serial measurements of complements were available had continuous hypocomplementemia as the state in which at least one of C3, C4, and CH50 were lower than the lower limit of the normal range in remission. In the other 4 patients, hypocomplementemia improved to normal or high levels of C3, C4, and CH50 in remission. There were no significant differences regarding clinical features at disease onset or in outcomes between the patients with continuous hypocomplementemia and those with normal complement levels.

## 4 Discussion

We evaluated the clinical significance of hypocomplementemia in AAV patients by analyzing their baseline clinical characteristics and prognoses, and our results indicated that the AAV patients with hypocomplementemia more frequently had DAH and TMA and had poor prognoses compared to the AAV patients without hypocomplementemia.

Complement is a part of the innate immune system that can eliminate pathogens. Activation of the complement system is also involved in the pathogenesis of systemic autoimmune diseases. Three pathways can cause C3 activation: classical, alternative, and lectin pathways.^[15]^ C3 activation leads to the activation of C5 to form the membrane-attack complex (MAC) C5b-9. A MAC can cause cell lysis.^[16]^

Complement has been shown to be associated with the pathogenesis of AAV. For example, activation of the alternative complement pathway is required to induce vascular inflammation in AAV.^[4]^ In renal tissue of AAV patients with positive MPO-ANCA, C3d, and factor B co-localized with MAC, the involvement of an alternative pathway of complement system was thus suggested.^[17]^ In addition to roles of MAC, it is reported that C5a mediates ANCA-induced glomerulonephritis^[18]^ and that C5a receptor blockade protected against MPO-ANCA-induced glomerulonephritis in a murine model.^[19]^

The reported incidence of hypocomplementemia in AAV in the Japanese population is 4.2% to 14.8%.^[20]^ Molad et al^[6]^ reported that 6 of 30 patients (20%) with MPO-ANCA-positive AAV had low C3 at disease onset. In our present study, 20% of the AAV patients had hypocomplementemia at disease onset. Low C3 was reported to be associated with a poor renal prognosis and life prognosis in a study of 6 MPO-ANCA-positive AAV patients,^[6]^ but the present investigation is the only study regarding clinical features of AAV with hypocomplementemia. Our present findings revealed life-threating organ damage in AAV patients with hypocomplementemia, but we did not observe poor renal prognoses among these patients.

This study suggests poor prognoses in patients with hypocomplementemia. To our knowledge, this is the first study revealing that AAV patients with hypocomplementemia had higher incidences of DAH and TMA, and that hypocomplementemia at disease onset is thus a poor-prognosis factor in AAV.

The reported incidence of DAH in AAV is between 8% and 36%.^[21]^ Hirayama et al^[20]^ noted that the incidence of DAH in AAV in a Japanese population was 15.4%. In our study, 14% of the patients had DAH. DAH can be caused by pulmonary capillaritis, and circulating and/or tissue deposits of immune complexes have been demonstrated in many of the diseases that cause pulmonary capillaritis.^[22]^ Because activation of the pathway of the complement system can be caused by immune complexes, hypocomplementemia would be caused by an excessive activation of complement pathways. The relationship between hypocomplementemia and DAH in our study may be explained by these facts.

Haas et al^[2]^ reported that 68 (54%) of 126 patients with ANCA-associated crescentic glomerulonephritis had immune complex deposits in renal biopsy specimens. In our present study, 38% of the AAV patients had immune complex deposits in renal biopsy specimens, and the AAV patients with hypocomplementemia had significantly more immune complex deposits in renal biopsy specimens than the AAV patients without hypocomplementemia. In addition, the patients with hypocomplementemia had high urine protein levels (2.24 g/gCr). Fujimoto et al^[23]^ reported that 13 patients with MPO-ANCA-associated glomerulonephritis had 1.2 (0.6–2.0) g/gCr (median interquartile range]) of urine protein. This result may support our hypothesis that immune complexes have an important role in the pathogenesis of AAV with hypocomplementemia.

Although it is already convincing that ANCA primarily elicits the pathologic process of AAV,^[3]^ according to our literature search, the titer of ANCA at disease onset does not correlate with the clinical severity of AAV or BVAS at disease onset.^[24]^ In the present study, we also did not observe a significant difference in the titer of ANCA at disease onset between the AAV patients with hypocomplementemia and those without hypocomplementemia. Therefore, it might not be denied that AAV patients with hypocomplementemia have an etiology that is different from that of AAV patients without hypocomplementemia. Conversely, hypocomplementemia may only reflect some ANCA-associated processes of complement activation.

Regarding TMA, Chen et al^[25]^ reported that of 220 patients with AAV excluding those with EGPA, 30 patients (14%) had concomitant renal TMA in a pathologic evaluation, and AAV with TMA was not rare. In contrast, only 4% of the AAV patients in our study had TMA. We may have overlooked some patients with TMA because of the difficulty in diagnosing TMA.

Manenti et al^[26]^ reviewed the cases of 29 patients with AAV with TMA and argued that mutations or risk haplotypes in genes encoding alternative complement regulatory proteins are associated with TMA. An excessive activation of an alternative pathway caused by a dysfunction of complement factor H and membrane cofactor protein, which can inhibit complement activation in normal conditions, will cause hypocomplementemia. In light of these concepts, hypocomplementemia may be associated with the occurrence of TMA. The significantly lower platelet levels observed in our study could be explained by TMA.

In addition to DAH and TMA, more skin lesions are seen in patients with hypocomplementemia. Sada et al^[27]^ reported that 39 of 156 patients with AAV (25%) had cutaneous manifestations. In our study, 50% of the patients with hypocomplementemia had skin lesions.

On the basis of the above discussion regarding hypocomplementemia in AAV, complement-based therapies are considered potentially effective. Eculizumab, a humanized monoclonal antibody that blocks the activation of terminal complement at C5 and prevents the formation of C5a and MAC, was effective in patients with paroxysmal nocturnal hemoglobinuria.^[28]^ An orally active small molecule that blocks C5a receptor 1 was also successful in sparing concomitant corticosteroids in Phase II studies of AAV patients.^[29]^ Various other complement-based therapies including C3-, factor B-, and factor D-targeted agents have been developed for many types of diseases. Complement-based therapies such as eculizumab treatment may be effective in patients with AAV whose cases are associated with dysfunctions of complement systems.

There are some limitations in this study. First, the patient series was too small to clarify the pathogenesis of hypocomplementemia, especially in each of the AAV types (EGPA, GPA, MPA, and RLV). The pathological findings from renal biopsies (which revealed the immune complex deposits) provided important information for pathogenesis, but the number of cases for which these findings were available was also too small. We therefore consider this study and its results as merely hypothesis-generating. Our findings must be tested in larger numbers of patients.

Second, the study was retrospective, and it is thus necessary to confirm the results by performing prospective studies. Third, the follow-up durations were short and it was difficult to evaluate precisely the initiation of dialysis and death. Fourth, we could not assess the clinical episodes including DAH and TMA after remission during the follow-up period. It is also important to assess the relationships between variables at the disease onset and the clinical episodes during the follow-up period.

Fifth, the study obtained only a limited amount of pathological data except for the immune complex deposits in the renal biopsy specimens. To confirm these findings, it is necessary to assess biopsy specimens from other organs such as lung, which was the site of lesions of more patients with hypocomplementemia compared to those without hypocomplementemia in this study. Finally, the results of this study must be interpreted with great caution because our patients were all Japanese individuals with AAV, the clinical manifestations of which differ between Japanese and Western populations; for example, more interstitial lung diseases and more positive MPO-ANCA were observed in studies of Japanese AAV patients.^[27]^ Our findings may not apply to AAV patients in Western countries.

## 5 Conclusion

Our results suggest that hypocomplementemia in AAV might become a risk factor for the development of DAH and TMA. Moreover, hypocomplementemia was revealed to be a poor-prognosis factor in AAV. It is thus very important to pay attention to the levels of complement at the diagnosis of AAV.

## Supplementary Material

Supplemental Digital Content
