# Supplementary figures and images for: Antineutrophilic cytoplasmic antibody-associated vasculitis with hypocomplementemia has a higher incidence of serious organ damage and a poor prognosis
Source: Medicine (Baltimore). 2016 Sep 16;95(37):e4871. doi: 10.1097/MD.0000000000004871 (PMC5402598; doi:10.1097/MD.0000000000004871)

Supplementary Figure


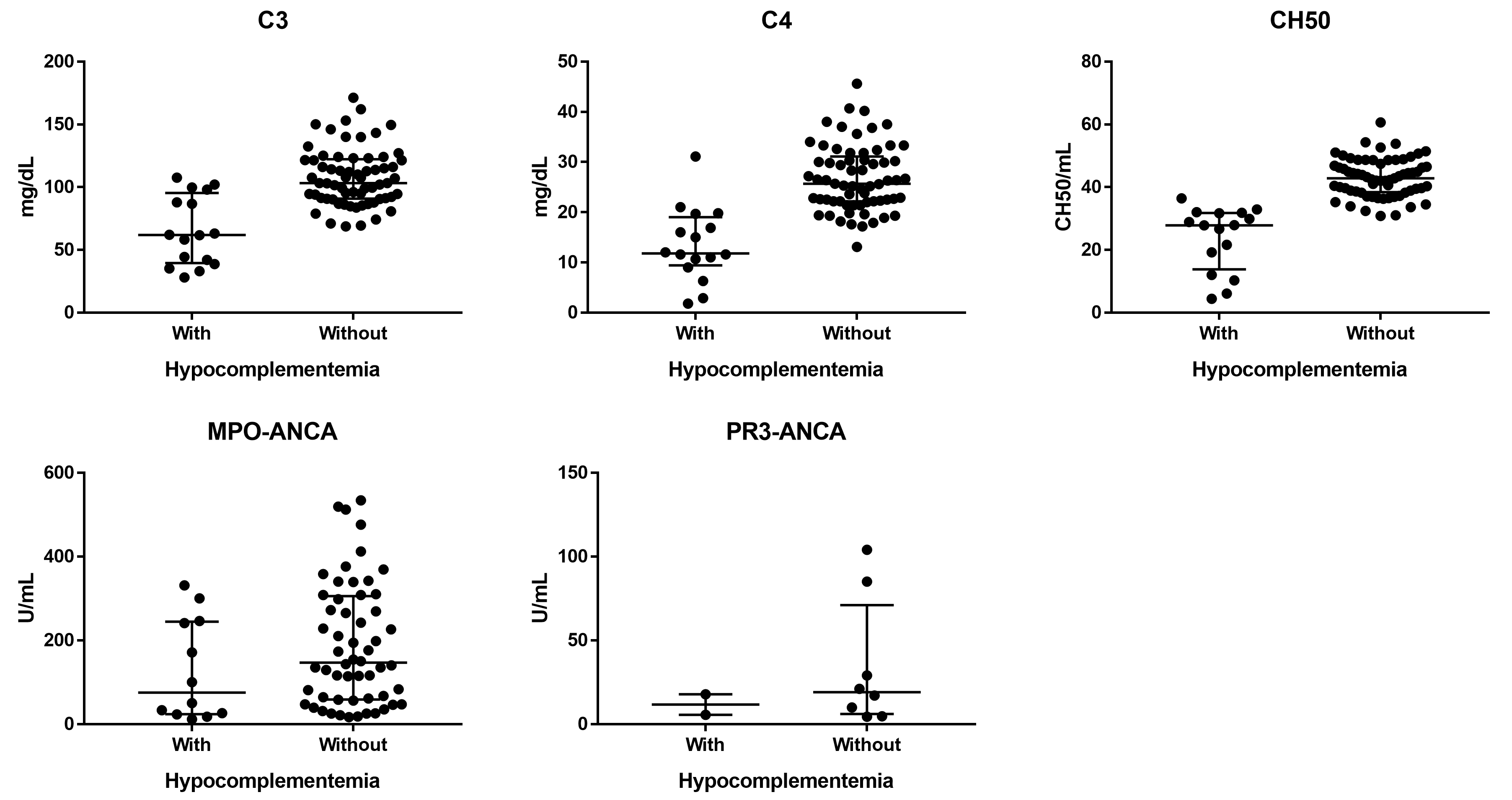

Supplement: Supplemental Digital Content [file medi-95-e4871-s001.doc]
